# Mechanistic Landscape and Farm to Fork Control Strategies for *Streptococcus suis*: An Emerging Foodborne Threat

**DOI:** 10.1155/tbed/1603643

**Published:** 2026-06-05

**Authors:** Wangyuan Yao, Meili Chen, Hongkai Ren, Qingqing Luo, Xuyuan Wang, Md. Akhtar, Zeeshan Ahmad Bhutta, Lianci Peng, Muhammad Yasir Nawaz, Mohammad Mehedi Hasan, Md. F. Kulyar, Rendong Fang

**Affiliations:** ^1^ College of Veterinary Medicine, Southwest University, Chongqing, 400010, China, swu.edu.cn; ^2^ College of Animal Science and Technology, Ningxia University, Yinchuan, Ningxia, 750021, China, nxu.edu.cn; ^3^ Institute of Clinical Immunology and Allergology, First Faculty of Medicine, Charles University, Prague, Czech Republic, cuni.cz; ^4^ State Key Laboratory of Molecular Vaccinology and Molecular, Diagnostics and Center for Molecular Imaging and Translational Medicine, School of Public Health, Xiamen University, Xiamen, 361002, China, xmu.edu.cn; ^5^ Department of Genetics and Genome Science, Michigan State University, Michigan, 48824, USA, msu.edu; ^6^ Department of Regenerative Medicine, State Research Institute Centre of Innovative Medicine, Vilnius, LT-08406, Lithuania

**Keywords:** biofilm-associated properties, blood–brain barrier, immune modulation, microbial-host dynamics, *Streptococcus suis*, zoonotic meningitis

## Abstract

*Streptococcus suis* (*S. suis*) is an emerging zoonotic agent that now rivals classical food‐borne pathogens in its global clinical burden of meningitis and septic shock. Recent epidemiological syntheses covering more than 30 countries now list over 1600 laboratory‐confirmed human cases with a pooled case‐fatality of about 12%, climbing beyond 18% in East Asia. The widely cited pooled case‐fatality estimate derives from a PRISMA‐guided systematic review and meta‐analysis that searched PubMed, Scopus, Web of Science, ScienceDirect, and Google Scholar through December 2012 and pooled study‐level event rates using inverse‐variance methods with random‐effects models when heterogeneity was present. We critically synthesize recent molecular, cellular, and translational studies to define how this swine commensal breaches host epithelial and blood–brain barriers (BBBs), subverts innate immunity, and disseminates systemically. Newly identified virulence mechanisms include serine–threonine kinase‐driven claudin‐5 cleavage, vimentin‐dependent transcytosis, quorum‐sensing control of biofilm maturation, and metabolic reprogramming that fuels neutrophil evasion. We integrate multiomics signatures with structural data to map conserved targets such as IdeSui and capsular polysaccharide that underpin next‐generation conjugate and nanoparticle vaccines. Diagnostic advances spanning CRISPR‐based assays and high‐resolution imaging are assessed for their capacity to enable point‐of‐care detection. We also highlight host transcriptomic signatures that can be integrated into microfluidic chips, allowing syndromic discrimination between *S. suis* and pneumococcal meningitis within 40 min, a critical window for targeted therapy. Finally, we present a prevention framework uniting farm biosecurity, rational antibiotic stewardship, probiotic and bacteriocin interventions, and community education. Collectively, the review delivers an up‐to‐date roadmap for mitigating *S. suis* transmission and disease, highlights outstanding knowledge gaps in host–pathogen interactions, and outlines translational priorities needed to transform bench discoveries into effective public health countermeasures.

## 1. Introduction

*Streptococcus suis* (*S. suis*) is a gram‐positive bacterium, and its pathogenicity causes significant economic losses in the swine industry and poses a serious threat to human health [1, 2], with $300 million in losses alone in the USA annually [3]. Human costs are smaller in absolute terms but substantial per case: Vietnam data estimated a mean direct cost of about US$1635 per episode, whereas Thai studies estimated average hospital treatment costs near US$4016 per admission and national productivity losses of about US$11.3 million annually. Stochastic farm‐level modeling across Germany, the Netherlands, and Spain projects an annual direct cost of 1.2 billion USD to the global swine sector, even before accounting for human medical expenses and productivity losses. *S. suis* is considered an occupational disease agent in humans [4]. Due to a higher zoonotic potential, *S. suis* has become a public health concern, threatening human health safety. Those working in the pig industry are particularly at risk [5]. Although meningitis has an infection rate of up to 84.6% and a high mortality rate and is the primary *S. suis*‐induced disease in humans, other complications include arthritis, peritonitis, pneumonia, septicemia, and endocarditis [4, 6]. Human *S. suis* infection is most commonly reported in Asia, but under‐recognition outside Asia is increasingly evident. Northern Togo surveillance identified 15 laboratory‐confirmed meningitis cases during 2010–2014, and later reports from Togo and Madagascar confirmed the circulation of zoonotic lineages in Africa [7]. Phylogeographic analyses reveal five dominant clades with distinct antimicrobial resistance islands, reinforcing the need for a One Health surveillance network that tracks both livestock and human isolates. Recent population genomics more securely supports several major clinically relevant lineages rather than an invariant set of five clades: CC1/ST1 strains are commonly serotype 2 or 14 and dominate zoonotic disease, ST7 marks hypervirulent outbreak‐associated strains, ST16‐centered lineages dominate many serotype 9 collections, and ST25/ST28 define distinct serotype 2 backgrounds in North America. Comprehensive resistome profiling of 742 isolates from Asia, Europe, and North America uncovered a 21 kb integrative conjugative element, ICESsuHN105, that colocalizes optrA, ermB, tetM, and lnuB, driving cross‐class resistance and pushing the modeled treatment‐failure risk above 40% for macrolides and lincosamides. Due to its exceptional colonization nature in the host GIT, even healthy pigs could carry several *S. suis* serotypes in the tonsils, genital organs, upper respiratory tract, nasal cavities, and GIT. *S. suis* commonly colonizes the palatine tonsils, and its mode of transmission is oral or nasal [8]. Thus, controlling the zoonotic potential of *S. suis* could be beneficial for pig production and end consumers. This review highlights the mechanism by which *S. suis* breaches the host blood–brain barrier (BBB). In particular, adhesion of *S. suis*, disruption of tight junction structure, invasion of endothelial cells following inflammation, penetration into the central nervous system (CNS) by breaking the BBB, evasion of host immune defenses, and, finally, invasion of the CNS, causing meningitis, are discussed. Significant preventive measures for controlling *S. suis* infection are also described.

## 2. Adhesion to Invasion: Insights Into *S. suis* Pathogenesis and Zoonotic Transmission Dynamics

**Figure 1:**
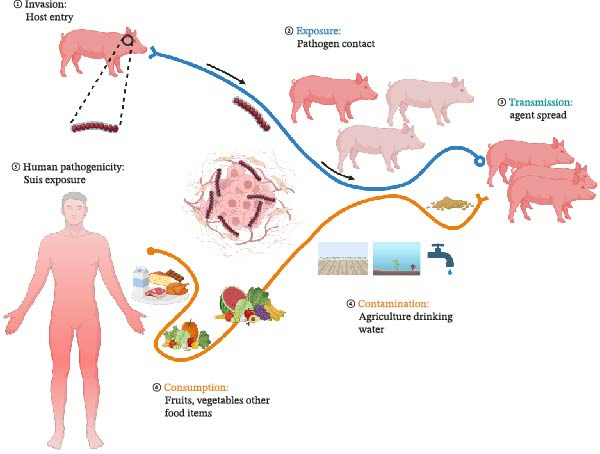
Schematic representation of the transmission dynamics of *Streptococcus suis*. The pathogen enters the porcine host and is then transmitted between animals, while environmental contamination occurs through agricultural practices and water. Human infection with this organism usually results from the consumption of contaminated food products, underscoring the zoonotic potential of *S. suis*.

For clarity, we group adhesion determinants by infection stages: early mucosal colonization, epithelial engagement, vascular persistence, and neurovascular interaction. We prioritize determinants with support from tissue or animal models, including sortase‐dependent surface proteins, SssP1, factor H‐binding protein, enolase, and capsule‐mediated immune evasion, and we treat lesser‐studied candidates as provisional rather than equivalent drivers. Several *S. suis* virulence factors, including adhesins, surface antigen 1, muramidase‐released proteins, capsular polysaccharides, hyaluronate lyase, suilysin, and extracellular proteins, have been implicated in clinical manifestations in humans and pigs and facilitate *S. suis* adhesion and invasion of host cells [7]. The fimbria‐like adhesin SssP1 was shown to bind to extracellular matrix components and to drive enhanced colonization of the porcine tracheal epithelium, broadening the catalog of invasion determinants. Before invasion, *S. suis* can colonize the respiratory tract and then invade via the respiratory epithelium or through the intestinal epithelium [9]. *S. suis* adherence is critical in successfully developing a carrier state in the host [10]. Serotypes (2 and 9) of *S. suis* consist of multimodal adhering proteins, i.e., antigen I and antigen II, which, through salivary glycoproteins, help *S. suis* to aggregate, adhere, and colonize in the pig’s upper respiratory system. These antigens facilitate biofilm formation, thereby protecting *S. suis* from low gastric pH [11]. After entering the bloodstream, the bacteria begin to penetrate the host brain by adhering to brain capillary endothelial cells and eventually crossing the BBB [12]. *S. suis* regulates different proteins to achieve successful adhesion through regulating two‐component regulatory systems (TCS), which are activated by the sensor kinase protein and induce or repress different genes [12, 13]. Further, revSC21, revS, and ciaRH encode orphan regulators, and knocking out these genes reduces adhesion to the Hep‐2 cells [14–16]. SalK/SalR is another TCS, and rgg and covR are the genes that help increase *S. suis* virulence [12]. AI‐2/LuxS is a quorum‐sensing system and an autoinducer that controls adhesion, biofilm formation, and virulence in *S. suis* [17]. Some cell adhesion molecules, along with genes such as SELE, CD34, SELP, SELL, VCAM‐1, ICAM‐2, and ICAM‐1, facilitate *S. suis* adhesion [18]. The sortase‐A gene of *S. suis* interacts with the host extracellular proteins and promotes *S. suis* adherence to type I collagen and cellular fibronectin [19]. The encapsulated *S. suis* resists uptake by macrophages and monocytes, indicating antiphagocytic properties that enhance *S. suis* adhesion. *S. suis* is also resistant to protease and performs the P‐adhesion process by recognizing a disaccharide sequence, Galα1‐4Gal, on the trihexosylceramide GbO3 [20, 21]. Streptococcal adhesin protein enhances *S. suis* adhesion to human intestinal epithelial cells, independent of the capsular polysaccharide, and significantly contributes to zoonosis [22]. These reports indicated that *S. suis* initiates pathogenesis by binding to and adhering to host cells and host proteins, thereby promoting virulence [23] (Figure 1).

## 3. Breaking Barriers: *S. suis*‐Mediated Disruption of Tight Junction Integrity and BBB Compromise

Tight junctions form a protective fence on epithelial cells, separating the basolateral and apical membranes and regulating paracellular permeability to support transepithelial transport. For penetration, *S. suis* applied to these epithelia, thereby moderating paracellular permeability for several vital substances [24]. *S. suis* induced immense rearrangements of different tight junction proteins, including claudin‐1, occludin, and zonula occludens‐1, and triggered the formation of basolateral stress fibers [24]. In addition to claudins, occludin, and ZO proteins, endothelial tight junctions also rely on junctional adhesion molecules such as JAM‐A and JAM‐C, which regulate paracellular sealing and leukocyte passage. Direct *S. suis*‐specific evidence for JAM dysregulation remains limited, so we treat JAMs here as a relevant knowledge gap rather than a confirmed mechanism. The vital mechanism by which *S. suis* exerts its pathogenicity is the disruption of claudin‐5, an essential element in maintaining BBB integrity [25]. Knockout of the serine/threonine kinase (STK) gene attenuates claudin‐5 cleavage in vitro and reduces brain bacterial counts by 10‐fold in a murine model, confirming STK as a druggable target. STK‐dependent claudin‐5 injury is a validated route of barrier disruption, but later work indicates that barrier outcomes are context‐dependent rather than uniformly irreversible. Human choroid‐plexus organoids supported *S. suis* translocation without overt barrier collapse, whereas host‐protective pathways such as dexamethasone‐sensitive junction preservation and EphA2‐linked autophagy suggest that barrier recovery mechanisms also merit discussion. Structure‐guided virtual screening has identified a benzimidazole analog, BZI‐45, that blocks STK autophosphorylation at 0.7 µM and rescues zebrafish embryos from lethal challenge, establishing the first small‐molecule lead for antivirulence therapy. Muramidase‐released proteins from *S. suis* compromise claudins at tight junctions, facilitating this bacterium’s traversal across microvascular endothelial cells in the cerebrum [25, 26]. The termini of two occludin‐like proteins, marvel‐D3 and tricellulin, are cytosolic, and their transmembrane helices are interconnected with two extracellular and one intracellular loops, resulting in MARVEL domain formation [27]. Upon *S. suis* invasion, the insoluble Triton‐X fraction was converted to a soluble Triton‐X fraction, which further induced degradation and dephosphorylation of the occludin protein [24]. *S. suis* also produces proteases that disrupt the integrity of tight junctions [28, 29]. The disruption of tight junctional proteins by porcine circovirus facilitates the translocation of *S. suis* in the respiratory epithelium [30]. An in vitro model showed that *S. suis* can invade a tight monolayer, and due to cell polarity, invasion is specifically observed in the epithelial cells of the choroid plexus [31]. The degradation of claudin‐5 is dependent on the expression of the STK gene [25]. When serotype 2 of *S. suis* was incubated with human intestinal epithelial cells, it could rearrange tight junctions only after 2 h of incubation and translocate in a paracellular manner [28]. *S. suis*‐induced disruption of tight junctions involves the production of inflammatory cytokines, bacterial adhesion, and oxidative stress. Each of the above‐mentioned processes compromises the integrity of the barrier through multiple pathways (Table 1).

**Table 1:** The Mechanisms of the disruption in tight junction integrity caused by *Streptococcus suis*.

Disruption mechanism	Description	Impact on tight junctions	Pathway involved	References
Cytokine production	Induces inflammatory cytokines leading to increased permeability	Weakens barrier integrity	NF‐κB signaling	[32, 33]
Adhesion and invasion	Bacterial adhesion to epithelial cells facilitates invasion	Disrupts tight junctions	Cell adhesion molecules	[23, 34]
Suilysin activity	Suilysin increases permeability by damaging endothelial cells	Compromises tight junctions	Cytotoxic effects	[35]
Neutrophil transmigration	Neutrophil infiltration exacerbates tight junction disruption	Further weakens barrier integrity	Inflammatory mediator release	[36, 37]

*Note:* This table shows a variety of processes that affect tight junctions and their associated signaling pathways.

**Figure 2:**
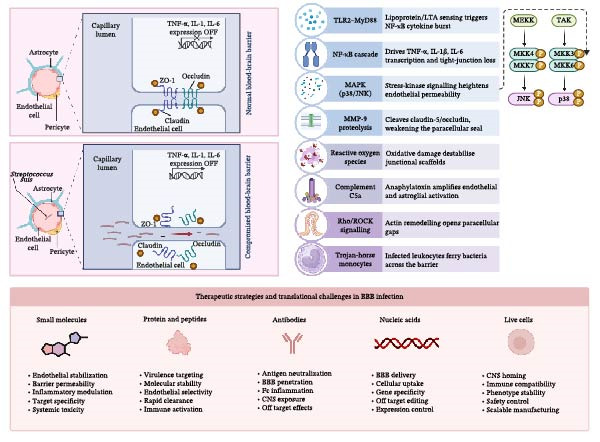
*Streptococcus suis*‐driven disruption of the blood–brain barrier (BBB) and associated therapeutic hurdles. The intact BBB illustration shows endothelial tight junctions formed by claudin, occludin, and ZO‐1 that seal the paracellular space, while transcription of TNF‐α, IL‐1, and IL‐6 remains repressed. In the adjacent compromised‐BBB diagram, *S. suis* contact triggers cytokine expression, disassembly of junctional strands, downregulation of junctional proteins, and increased paracellular leakage. A separate illustration of healthy intestinal epithelium underscores the shared reliance on tight junction architecture across barriers. An accompanying panel summarizes five therapeutic classes under investigation for central nervous system infections and the principal challenges associated with each.

*S. suis* possesses autolysins, which are glycosidases, peptidases, and amidases, cleaving the glycan backbone within the peptide side chain and the peptide side chain, respectively, increasing *S. suis* pathogenicity [38]. The above literature indicates that tight junction proteins are highly important to maintain barrier integrity, but *S. suis* could rearrange its proteins, disrupt tight junctions, and increase barrier permeability, facilitating its translocation through the barrier and promoting infection (Figure 2).

## 4. *S. suis* Induces an Inflammatory Response, Leading to Bacterial Distribution

*S. suis* has been reported as a substantial zoonotic pathogenic bacterium, which could induce the worst inflammatory response in humans and pigs. *S. suis*‐induced inflammation is a life‐threatening characteristic because it promotes microbial dissemination and contributes to host tissue damage and systemic diseases, particularly meningitis [39]. *S. suis*‐induced inflammatory response is triggered through the activation of macrophages and neutrophils. For example, IL‐1 significantly orchestrates the host immune response, particularly depending on the specific strain, and is intensely involved in inflammation and microbial clearance [39]. However, enthusiasm for broad IL‐1 blockade should be tempered as IL‐1 receptor antagonist trials in unselected sepsis populations have not shown a clear overall mortality benefit, highlighting the need for biomarker‐guided rather than indiscriminate cytokine targeting. The activation of IL‐1 signaling leads to the secretion of other cytokines, such as TNF‐α and IL‐6, thereby exacerbating the host inflammatory response [40]. *S. suis* induced inflammation by activating the MMP3 gene through extracellular signal‐regulated kinase phosphorylation and releasing TNF‐α [24]. *S. suis*‐secreted suilysin is a cytolysin, which is cholesterol‐dependent and has been reported to induce NLRP3 inflammasome activation. Suilysin should also be viewed in the broader context of meningitis‐associated pore‐forming toxins. Like pneumolysin and group B streptococcal β‐hemolysin/cytolysin, it couples membrane injury to cytokine amplification and barrier dysfunction, although the best direct evidence in *S. suis* still centers on suilysin itself. This pore formation simultaneously triggers gasdermin‐D cleavage and pyroptotic cell death, amplifying BBB leakage and bacterial dissemination. Downstream transcriptomics of infected microglia reveal a surge of interferon‐β and CXCL10 driven by cGAS‐STING activation, linking DNA‐sensing pathways to the neuro‐cytokine storm. Upon activation of the NLRP3 inflammasome, an overwhelming storm of IL‐18 and IL‐1β is triggered, further intensifying the inflammatory response and leading to streptococcal toxic shock‐like syndrome [41]. This condition could lead to severe clinical manifestations, such as different organ failures and septic shock [40]. The pore‐forming capability of suilysin could disrupt the cell’s integrity and cause cell lysis, thereby releasing damage‐associated molecular patterns [42, 43]. Capsular polysaccharide limits the activation and recruitment of host immune cells, thereby influencing the whole inflammatory response. Similarly, *S. suis*‐released lipoprotein promotes *S. suis* invasion, adhesion, infection, and finally, an inflammatory cascade [44]. Lipoproteins are also included among pathogen‐associated molecular patterns that trigger the toll‐like receptor signaling cascade, leading to the release of excess pro‐inflammatory cytokines [45]. Upon *S. suis* infection, MAPK and NF‐κB pathways are activated [39, 40]. Worryingly, *S. suis*‐induced neuroinflammation is attributed to VraSR, a two‐component signal‐transduction system implicated in the disruption of tight junction proteins and the dissemination of inflammation, contributing to resistance to the host innate immune defense [46, 47]. These reports revealed that *S. suis*‐induced infection or inflammation is a multifaceted process involving virulence factors, the immune signaling cascade, and the host immune response. The pathogenic characteristics of *S. suis* causing meningitis in humans are shown in Table 2 [48]. The “Trojan horse” entry in Table 2 summarizes predominantly experimental animal and in vitro evidence rather than direct human in vivo observations.

**Table 2:** The pathogenic characteristics of *S. suis*, causing meningitis in humans.

Parameter	Details	Additional notes
Pathogen	*Streptococcus suis* (*S. suis*)	Gram‐positive, zoonotic pathogen
Host age group	Adult	Primarily affects immunocompromised individuals
Adhesion and invasion mechanisms	Cytolysin, lipoteichoic acid, protein anchored in bacterial cell wall, capsule	Key factors in host interaction and immune evasion
Entry site or colonization	Cutaneous wound, nasopharynx, intestine	Common routes of infection in both humans and animals
Survival mechanisms	Complement inhibitors, capsule‐dependent protection	Essential for evading host immune response
Dissemination	Monocytes and macrophages via Trojan Horse pathway	Facilitates spread within the host
Entrance mode into the CNS	Breaking the blood–brain barrier (BBB) or blood‐cerebrospinal fluid barrier	Critical step leading to severe neurological symptoms
Clinical manifestations	Meningitis, peritonitis, sepsis, arthritis, endocarditis, pneumonia, Streptococcal septic shock‐like syndrome	Highly variable, depending on infection site and host response
Etiology of CNS tissue damages	Enhanced permeability of BBB, increased levels of inflammatory cytokines	Leads to neurological dysfunction and severe complications

## 5. The Inflammatory Cascade: Mechanisms of Pathogenesis and Neuroinflammation

**Figure 3:**
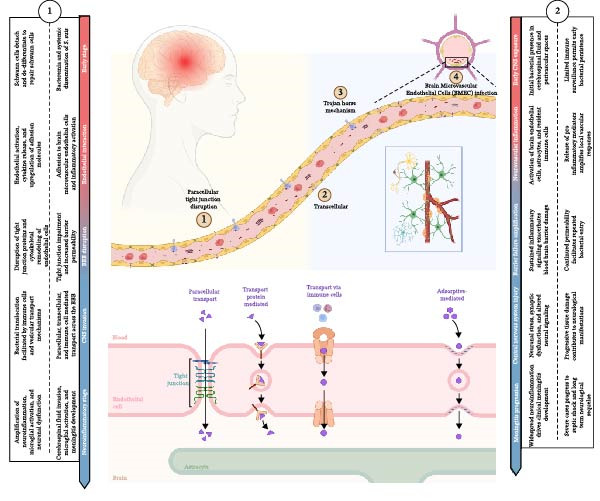
*Streptococcus suis* crossing the blood–brain barrier. This is the mechanism by which *S. suis* breaks the blood–brain barrier and penetrates the cerebrospinal fluid barrier. *S. suis* uses paracellular transport, protein‐mediated transport, transport via immune cells, and adsorptive‐mediated transport.

The BBB is a dynamic protective barrier that safeguards the CNS from microbial‐produced toxins. However, BBB permeability is significantly compromised during infectious conditions, allowing the entry of pathogenic and harmful microbes into the CNS [49]. Disruption of the BBB can lead to serious neurological consequences, including meningitis [25]. The penetration of *S. suis* to the BBB is governed by transcytosis, transcellularly, paracellularly, and Trojan horse mechanisms [50, 51]. Transcytosis is the endocytosis of microbes or substances across the endothelial cell barrier, which maintains CNS homeostasis, allows the selective transport of signaling molecules and nutrients, and prevents the entry of harmful microbes [52]. In transcytosis, endothelial cells take up *S. suis* via receptor‐mediated endocytosis, transport it across the cells, and release it into the CNS. *S. suis* internalization is achieved after adhering to the brain microvascular endothelial cells. *S. suis* penetration ability into the BBB has been enhanced. *S. suis*‐produced serine/threonine protein kinase follows by modulating claudin‐5, whose disruption further promotes transcytosis of *S. suis* across the brain endothelial barrier [9, 25]. *S. suis* interacts with vimentin and encourages *S. suis* transcytosis into the BBB, promoting bacterial access to the host CNS [53]. In the paracellular passage, *S. suis*, through its muramidase‐released protein, degrades tight junction proteins and the extracellular matrix, thereby increasing barrier permeability [2, 54]. Certain pathogens utilize the Trojan horse mechanism to evade the host immune response and gain access to the host CNS by hitching a ride on host immune cells [55]. A Trojan horse route remains biologically plausible but has not yet been directly visualized in vivo for *S. suis*. Current support derives mainly from barrier coculture models, blood‐CSF barrier studies, and animal infection systems rather than direct human in vivo imaging. Single‐cell RNA sequencing of infected brain microvascular endothelial cells shows upregulation of LAMP2 and caveolin 1 during this process, suggesting a shift toward caveola‐mediated transcytosis. In this process, *S. suis* uses host immune cells to penetrate the BBB. During bacteremia, *S. suis* binds to the host monocytes and breaches the BBB, or blood‐cerebrospinal fluid barrier, through these cells via D‐alanylation of LTA [3, 9, 56]. *S. suis*‐induced inflammation enhanced the expression of adhesion substances, which expedited the transmigration of the host immune cells that carry pathogenic bacteria [37]. *S. suis* was found in the infected individual’s CNS, indicating the traversing of *S. suis* across the BBB and establishing an infection [57]. A noteworthy upsurge in macrophages and neutrophils serves a dual function in *S. suis* infection, exacerbating inflammation, facilitating bacterial transport across the BBB, and increasing meningitis pathogenesis [58]. These studies indicate that Trojan horse and transcytosis are vital mechanisms that enable *S. suis* to cross the host BBB and establish infection within the host CNS. There are different pathways, such as clathrin‐mediated endocytosis and phagocytosis, which are important specific pathways involved in this barrier compromise during infection (Table 3). Real‐time two‐photon intravital imaging has captured paracellular breach events that precede detectable tight junction loss by 8 minutes, suggesting a rapid gating mechanism that remains mechanistically unresolved. This ability of *S. suis* to manipulate the host’s immune response poses a new challenge for treating this harmful infection (Figure 3).

**Table 3:** A comparison of the mechanisms of penetration across the BBB by *Streptococcus suis*, including both transcytosis and Trojan horse strategies.

Mechanism of penetration	Host cells involved	Pathways utilized	Impact on BBB integrity	References
Transcytosis	Endothelial cells	Clathrin‐mediated endocytosis, caveolin‐mediated endocytosis	Moderate disruption; transient increase in permeability	[32, 33]
Trojan horse mechanism	Neutrophils, macrophages	Phagocytosis, immune evasion strategies	Significant disruption; prolonged permeability changes	[23, 34]
Direct infection of endothelial cells	Brain microvascular endothelial cells	Activation of signaling pathways (e.g., NF‐κB)	Severe disruption; loss of tight junction integrity	[35, 59]
Cytokine release	Astrocytes, microglia	Cytokine signaling pathways (e.g., IL‐1β, TNF‐α)	Increased permeability and inflammation	[36, 60]
Oxidative stress induction	Endothelial cells, astrocytes	ROS production, activation of stress response pathways	Compromised tight junctions, enhanced paracellular transport	[37]
Infiltration of immune cells	Monocytes, lymphocytes	Chemokine signaling (e.g., CCL2, CXCL8)	Increased BBB permeability due to inflammatory response	[23, 61]
Endothelial cell activation	Endothelial cells	NF‐κB and MAPK pathways	Disruption of tight junction proteins (e.g., occludin, claudin)	[23, 61, 62]
Microglial activation	Microglia	Cytokine and chemokine signaling	Enhanced neuroinflammation, leading to BBB disruption	[23, 61]
Alteration of lipid rafts	Endothelial cells	Disruption of lipid raft integrity	Impaired signaling and increased permeability	[62]
Matrix metalloproteinase activation	Endothelial cells, astrocytes	MMP signaling pathways	Degradation of extracellular matrix components, leading to BBB breakdown	[62, 63]

*Note:* This table further provides detailed information on host cells, involved pathways, and the degree of impairment in BBB integrity for each mechanism.

## 6. Immune Evasion and Biofilm Resilience: Strategies in Host Immune Modulation and Virulence

The host‐*S. suis* interaction seems more complex, as *S. suis* could escape the host immune detection mechanism and manipulate its cell signaling cascades, facilitating *S. suis* translocation through the BBB [37, 53]. The formation of polysaccharide capsules by *S. suis* is considered a primary strategy to evade the host immune system and acts as a physical barrier, inhibiting immune cell‐mediated phagocytosis. IgM can kill *S. suis*; however, the capsule protects *S. suis* and resists antibody‐mediated opsonophagocytic killing [3, 64]. In an early infection stage, the capsule of *S. suis* acts as a vital virulence factor, enabling it *to* survive in the host bloodstream [65]. In humans, complement factor H regulates complement activation, which is important for phagocytosis/opsonization. *S. suis* produces factor H‐binding proteins and inhibits the activation of the complement system by binding to complement factor H, efficiently evading phagocytosis and opsonization. *S. suis* utilizes this strategy in navigating the host immune defense, particularly in meningitis [66]. Additionally, M‐related protein helps *S. suis* resist phagocytosis, complicating the immune response by interfering with opsonization [67].

Static microtiter assays provide useful relative biomass rankings, but in vivo pig models show spatially restricted tissue aggregates rather than directly comparable bulk biomass values. We therefore interpret in vitro density primarily as a comparative laboratory phenotype rather than a direct proxy for tonsillar burden. *S. suis* also utilizes biofilm formation in evading host immune defense, which allows *S. suis* to live a resilient lifestyle and enables the bacterium to survive in a hostile environment [9]. Biofilm helps *S. suis* survive longer in the host through an intricate mechanism of adhesion, invasion, and dissemination, thereby developing multicellular communities. Phage‐based control and CRISPR‐enabled targeting remain early‐stage strategies for *S. suis*. Current evidence supports feasibility research, but strain coverage is incomplete, and bacterial antiphage defenses may limit durability, so we avoid presenting these approaches as established antibiofilm therapies. Initially, *S. suis* applies polysaccharides and surface proteins to adhere to the host abiotic surface. After adhering, it proliferates and produces an extracellular matrix that comprises extracellular DNA, proteins, and polysaccharides, providing essential structural integrity and protecting *S. suis* from environmental stressors, including antibiotics and immune response [68, 69]. A serotype 2 *S. suis* mutant with impaired capsule expression increased biofilm formation [69, 70]. Carbohydrate metabolism is upregulated during *S. suis* infection, providing the necessary precursors and energy for biofilm matrix production, indicating how *S. suis* influences metabolic pathways [71]. Epigallocatechin gallate suppresses biofilm formation by downregulating capsular polysaccharide proteins and decreasing bacterial adhesion [72]. Several genes are involved in *S. suis*‐produced biofilm formation, regulating matrix constituents and surface proteins, such as the gene that encodes type‐I restriction‐modification protein HsdS, which is linked to surviving inside phagocytes and biofilm formation, and the gene related to FtsA, a cell‐division protein, is vital in developing a biofilm [73]. Reported pro‐biofilm effects are strain‐dependent: amoxicillin increased biofilm at 1/2–1/4 MIC, lincomycin at 1/2–1/32 MIC, and oxytetracycline at 1/4–1/16 MIC in ISU1606, whereas 1/4 MIC amoxicillin and 1/4 MIC tylosin increased biofilm in HA9801; for that strain, 1/4 MIC corresponded to 0.078125 μg/mL and 0.3125 μg/mL, respectively [74, 75]. The presence of *Actinobacillus pleuropneumoniae* with the biofilm enhanced antibiotic resistance and modulated the expression of virulence factors [72, 76]. For example, biofilms provide a safe niche for microbial cells, allowing them to evade immune responses and resist antibiotics. The development of neutrophil extracellular traps is essential for trapping and killing pathogenic bacteria. At the same time, *S. suis*‐produced biofilm resists neutrophils’ trapping, killing, and phagocytosis, enhancing survival ability and developing chronicity in *S. suis* infections [77]. Collectively, *S. suis* uses immune modulation, surface protein expression, genetic diversity, capsule formation, and biofilm formation to evade the host immune response. These approaches promote *S. suis* virulence and infection in humans and swine. *S. suis* virulence factors are shown in Table 4 [78].

**Table 4:** *Streptococcus suis* virulence factors.

Category	Virulence factor	Function/target
Virulence factors	Hyaluronate lyase	Degrades hyaluronic acid, targeting brain microvascular endothelial cells
	Type IV secretion system	Facilitates interaction with host cells
	SspA (serine‐associated subtilisin‐like protease)	Acts as a peptidyl isomerase
	HP171	Specific virulence factor of *Streptococcus suis*
	Amylase‐binding protein B	Binds to red blood cells (RBCs) and endothelial cells, leading to inflammation and cytokine production
	Suilysin	Damages host cells, particularly RBCs
Cell surface proteins	Enolase	Binds to plasminogen, promoting extracellular matrix degradation
	Sortase	Anchors proteins to the cell surface
	Autolysin	Facilitates cell wall remodeling.
	GADPH	Also binds plasminogen
	Opacity factor	Modulates interactions with host cells
	Extracellular factor	Promotes adhesion and invasion
	Oligopeptide permease	Contributes to nutrient acquisition
	Histidine triad protein C	Plays a role in host–pathogen interaction
	Streptococcal adhesion P	Facilitates binding to host cells
	Muraminidase‐released protein	Targets fibronectin and fibrinogen
	Fibronectin/fibrinogen binding protein	Interacts with the extracellular matrix (fibronectin, laminin, and collagen)
Capsule‐associated factors	EndAsuis	Interacts with human factor B.
	DltA	Galanylation of lipoteichoic acids
	SntA	Surface‐exposed heme‐binding protein
	Pgd	Acts on peptidoglycan
	SsnA (exonuclease)	Avoids neutrophil extracellular traps (NETs)
	Dipeptidyl peptidase	Contributes to immune evasion

*Note: S. suis* **-** produced degradative enzymes and toxins facilitate its dissemination by releasing several pro‐inflammatory cytokines, which not only cause meningitis in humans and pigs but also lead to other associated systemic diseases. *S. suis* releases different kinds of adhering and surface proteins, enabling *S. suis* in adhesion, invasion, and dissemination. These proteins also help *S. suis* form the extracellular matrix, further enhancing its pathogenesis. *S. suis* can produce those factors that can degrade host immune components and resist phagocytic killing, prolonging its survival in the host [78].

## 7. Comprehensive Strategies for Preventing Infections: Biosecurity, Vaccination, and Antimicrobial Management

*S. suis* poses a serious health risk to both humans and pigs. The prevention of *S. suis* infections could involve prudent antimicrobial use, appropriate vaccination strategies, and biosecurity measures [79]. Active biosecurity procedures are necessary to prevent *S. suis* transmission in pigs and humans. To this end, environmental contamination could be reduced by following standard hygienic measures, limiting access to livestock farms, and applying appropriate management practices [3, 80]. To identify early outbreak risks, continuous monitoring of different *S. suis* strains in pig farms is necessary [81]. Further, close contact between humans and pigs must be reduced in slaughterhouses and animal farms to minimize zoonotic transmission [82, 83]. Vaccination is another main component in preventing *S. suis* infection. Different vaccines are administered to boost immune defense against different *S. suis* serotypes [84, 85]. A multicomponent subunit vaccine combining enolase, GAPDH, and sodium‐binding protein Sbp conferred complete protection against lethal heterologous challenge in piglets and is entering field trials. Complementary immunoproteomic screens have now added PdhA, Ldh, and MalX; inclusion of these antigens boosts TH17 polarization and achieves sterilizing immunity in murine intranasal models, paving the way for a hexavalent universal vaccine. These vaccine concepts remain promising, but serotype 9 isolates exhibit marked genomic and virulence‐associated diversity, so cross‐protection against serotype 9 cannot yet be assumed and should be explicitly validated in future challenge studies. The *S. suis* immunogenic component, Ide *S. suis*, an enzyme to degrade immunoglobulin M, which helps develop an effective vaccine [84]. The prudent use of antimicrobial compounds is critical for managing *S. suis* infection, particularly as many bacteria have become resistant to the antibiotics currently used to treat bacterial infections, complicating treatment [86, 87]. Promoting biosecurity measures, responsible antibiotic use, and the adoption of alternative therapies could decrease the risk [86, 87]. Since 2022, European regulations have ended prophylactic group treatments and banned medicinal zinc oxide, accelerating the shift toward precision‐microbiome and vaccine‐only approaches in pig production. Developing bacteriocins from nonvirulent *S. suis* strains could be promising for alternative therapies. For example, suicin 3908 is an *S. suis*‐produced lantibiotic isolated from healthy pigs and exhibits antimicrobial activity against almost all *S. suis* pathogenic strains [88, 89]. Workers at pig farms or in meat processing companies are at higher risk of *S. suis* infection. Wearable sensor platforms that fuse subtle body‐temperature drift with cough‐acoustic signatures now flag early disease clusters among abattoir staff with 90% precision, offering a proactive occupational health sentinel. These workers must be aware of the *risks associated with S. suis* infections [82, 83]. Additionally, educational programs should be implemented to guide these workers on meat‐handling procedures, health and safety measures, the use of personal protective equipment, and the symptoms of *S. suis* infection [82, 83]. Thus, *S. suis* infection could be prevented by implementing comprehensive strategies, including antimicrobial stewardship, vaccination, appropriate biosecurity measures, and raising public health awareness. The *S. suis* infection risk could be considerably decreased in humans and pigs by following these comprehensive approaches.

### 7.1. Prevention and Treatment Prospects Through Intestinal Flora and the Gut–Brain Axis

The connection between the gut microbiome and the CNS—the gut–brain axis—is an aspect that has received unprecedented attention in the case of infectious diseases caused by *S. suis*, especially in the recent era. This is generally viewed as a swine pathogen but in later years has been recognized as a significant zoonotic threat to humans, leading to grave diseases such as meningitis and septicemia [3, 90]. This offers promising perspectives for therapeutic interventions aimed at understanding the mechanisms by which the gut microbiota may contribute to the prevention and control of *S. suis* infection through the gut–brain axis. The gut microbiota is therefore pivotal in regulating the immune system, which, in turn, is indispensable for combating infections, including those caused by *S. suis*. A balanced microbiome can enhance the host’s immune response, potentially reducing the severity of infections [91, 92]. Probiotics, live microorganisms that confer health benefits, have been shown to influence the gut microbiome positively and, consequently, the immune system. *Lactobacillus plantarum* strain WCFS1, engineered to secrete bacteriocin suicin 3908, reduced tonsillar *S. suis* carriage by 2 log units in weaned piglets, illustrating an adjunct gut–brain axis intervention. Metagenome‐wide association studies further link enrichment of short‐chain fatty acid producers, such as *Faecalibacterium prausnitzii* and *Roseburia* spp., with tighter intestinal and BBBs, pointing to prebiotic fiber as a synergistic adjunct. For example, some members of the genus *Lactobacillus* may enhance the production of anti‐inflammatory cytokines. These may diminish the inflammation that characterizes *S. suis* infections [93]. Additionally, the intestinal microbiota modulate neurotransmitter synthesis and a range of other signaling molecules that may affect cerebral function and behavioral patterns, thereby influencing the host’s overall health [92, 94]. This interaction suggests that maintaining a healthy gut microbiome is a potentially effective preventive strategy for avoiding infection by modulating the immune response and dampening inflammation in the CNS [95]. The brain–gut axis is also implicated in the pathogenesis.

The bacterium must traverse the BBB to induce meningitis, and this mechanism may be affected by the state of the gut microbiome [34, 37]. Dysbiosis, characterized by an imbalance in the gut microbiota, can increase BBB permeability, thereby promoting the entry of pathogens such as *S. suis* into the CNS [96]. Thus, approaches that restore proper gut flora hold promise for improving BBB integrity and reducing the risk of CNS infections. Certain nutritional factors, such as prebiotics, have been reported in the literature to enhance the proliferation of beneficial gut flora, which, in turn, would strengthen the gut–brain axis, thereby possibly reinforcing the integrity of the BBB against pathogenic invasion [97].

## 8. Conclusions

*Streptococcus suis* is an important zoonotic pathogen that combines adaptability, evasion, and penetration of host defenses with serious consequences for human and animal health. Due to its ability to breach the BBB via mechanisms such as adhesion, proteolytic disruption of tight junctions, and immune evasion, *S. suis* has become a major cause of meningitis and systemic infections. Its ability to induce a strong inflammatory response through multiple pathways, including NLRP3 inflammasome activation, MAPK signaling, and NF‐κB, suggests that this pathogen may manipulate host cellular mechanisms to increase tissue damage and promote disease progression. Moreover, *S. suis’*s ability to form a biofilm and synthesize a capsule indicates its resistance to the host’s immune response and its capacity to survive under hostile conditions. Addressing the threats posed by *S. suis* infection requires an integrated, interdisciplinary approach that considers enhanced vaccination programs, antimicrobial therapies, and biosecurity measures. Public awareness and education about zoonotic risks will be necessary for reducing the rate of human infection, especially among people with high occupational risk. This review describes the molecular basis of *S. suis* pathogenesis and provides a framework for developing targeted therapeutic approaches and preventive measures. Overcoming the challenges posed by *S. suis* requires a collective effort to protect animal and public health from this global threat. We advocate an integrated digital surveillance ecosystem that combines pathogen genomics, pig movement data, and environmental signals, with the measurable aim of shortening outbreak detection time and accelerating targeted farm and occupational health interventions. Future work should integrate multiomics host–pathogen interaction maps with artificial intelligence‐guided drug discovery pipelines to prioritize conserved fungal‐like metabolic chokepoints for narrow‐spectrum therapeutics.

## Author Contributions

Wangyuan Yao provided the idea and wrote the initial draft. Md. F. Kulyar and Zeeshan Ahmad Bhutta was responsible for the development of the figures, contributed to the critical revision of the manuscript, and played a key role in shaping the final draft. Meili Chen, Hongkai Ren, Qingqing Luo, Md. Akhtar, Muhammad Yasir Nawaz, Xuyuan Wang, and Mohammad Mehedi Hasan helped to analyze and evaluate the data. Lianci Peng provided the technical support. Rendong Fang supervised the study and revised the final draft of the manuscript.

## Funding

This work was supported by the National Natural Science Foundation of China (Grant 32473027), the Chongqing Science and Technology Commission (Grant CSTB2025NSCQ‐GPX0495), the Fundamental Research Funds for the Central Universities (Grants SWU‐KQ25019 and SWU‐XJPY202305), the Chongqing Modern Agricultural Industry Technology System (Grant CQMAITS202512), and the Co‐construction Project of Fuling Academy of Southwest University (Grant FLYJY202506).

## Disclosure

All the authors contributed to the final revision of the article and approved the submitted version.

## Conflicts of Interest

The authors declare no conflicts of interest.

## Data Availability

The data sharing is not applicable to this article, as no datasets were generated or analyzed during the current study.
